# Supplementary material for: Discovery of a sulfotyrosine-motif in the human TrkB extracellular domain required for agonist activation
Source: bioRxiv. 2026 May 21:2026.05.19.725324. Preprint. [Version 1] doi: 10.64898/2026.05.19.725324 (PMC13228283; doi:10.64898/2026.05.19.725324)
Supplement: Supplement 1 [file media-1.pdf]

Supplementary Materials for:

Discovery of a sulfotyrosine-motif in the human TrkB extracellular domain required for agonist activation

David C. Briggs[1], Ryan T. Duffy[2], Sarah Ateaque[3], Sarah Maslen[4], Hema Nagaraj[5], Yves-Alain Barde[3], Peter S. DiStefano[6], Ronald M. Lindsay[6], Paul C. Armstrong[7], Chloe J. Peach[2], Neil Q. McDonald[1,8] *

Corresponding author: neil.mcdonald@crick.ac.uk

The PDF file includes:

Figs. S1 to S6
Tables S1 to S2

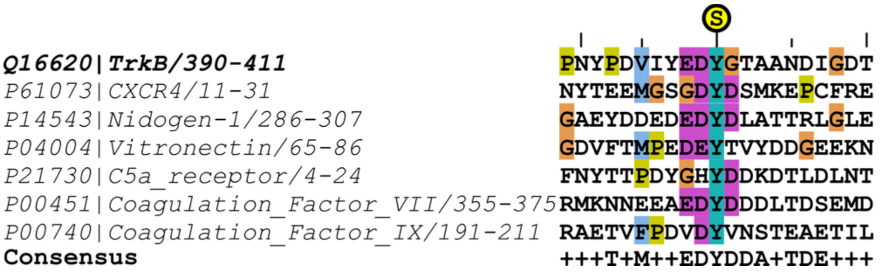

FigS1

Similarity of the TrkB sTyr400 motif to known sTyr-containing motifs found in selected human proteins. Uniprot identifiers and sequence extent are indicated.

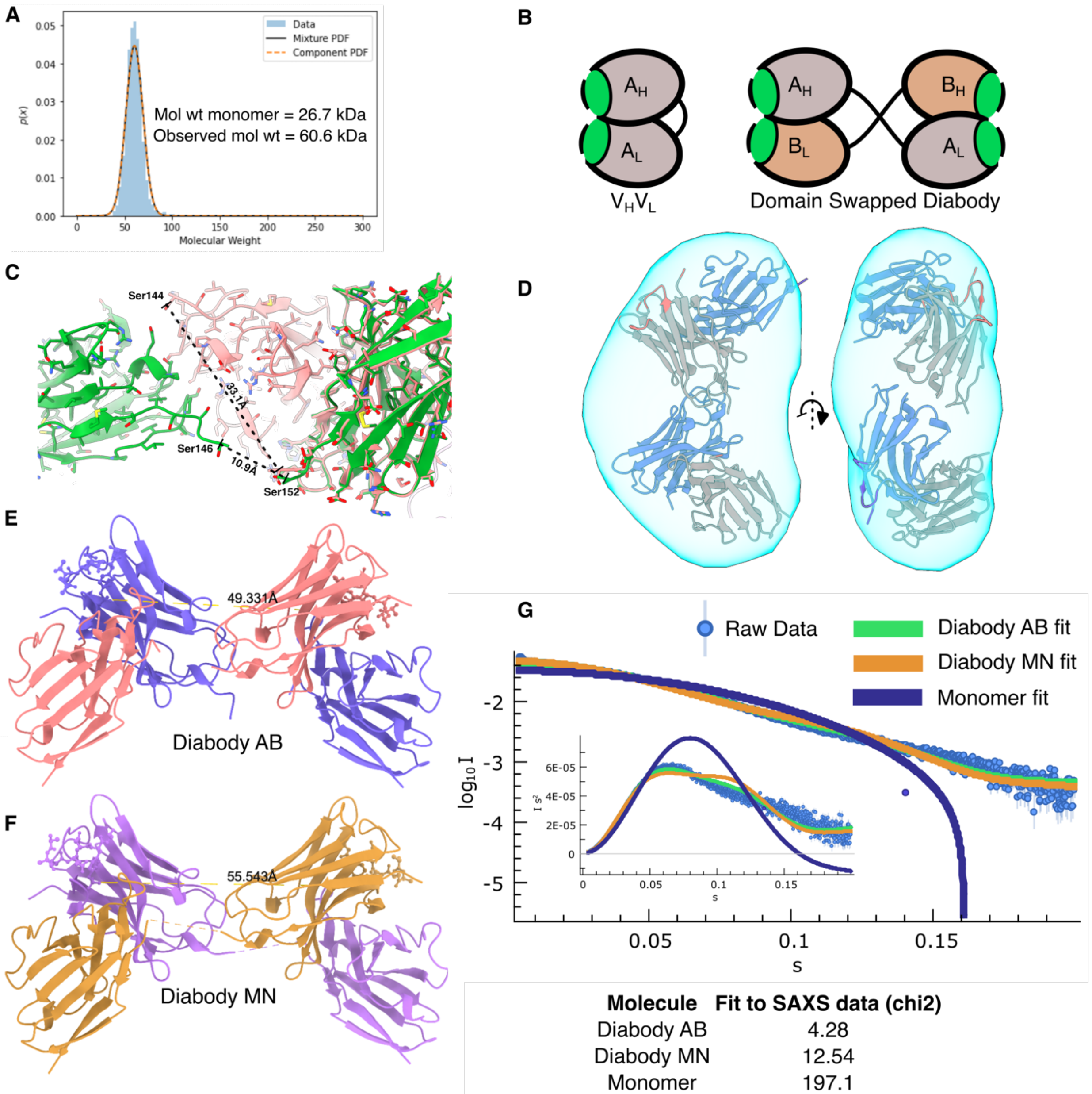

Molecule	Fit to SAXS data (chi2)
Diabody AB	4.28
Diabody MN	12.54
Monomer	197.1

Fig. S2.

Crystallographic and in solution evidence that ZAB85 adopts a dimeric state consistent with a diabody. (**A**) Mass photometry analysis of ZAB85 is consistent with a dimeric oligomeric state even at low nanomolar concentrations, (**B**) Schematic of a monomeric compared to a domain-swapped diabody influenced by connecting linker length. (**C**) The disordered linker distance in the domain swapped, P1 model (green, missing residues 147-151, 10.9 Å) is plausible, whereas the monomeric configuration (pink, missing residues 145-151, 33.1 Å) is not. (**D**) Ab-initio SAXS DAMMIN envelope is consistent with a diabody configuration. (**E,F**) the two independent diabodies observed from the P1 cell processed crystal structure. The sTyr-sTyr binding site distances are indicated. (**G**) Diabody AB is most consistent with the SAXS data, although the Kratky plot (inset) is indicative of some flexibility.

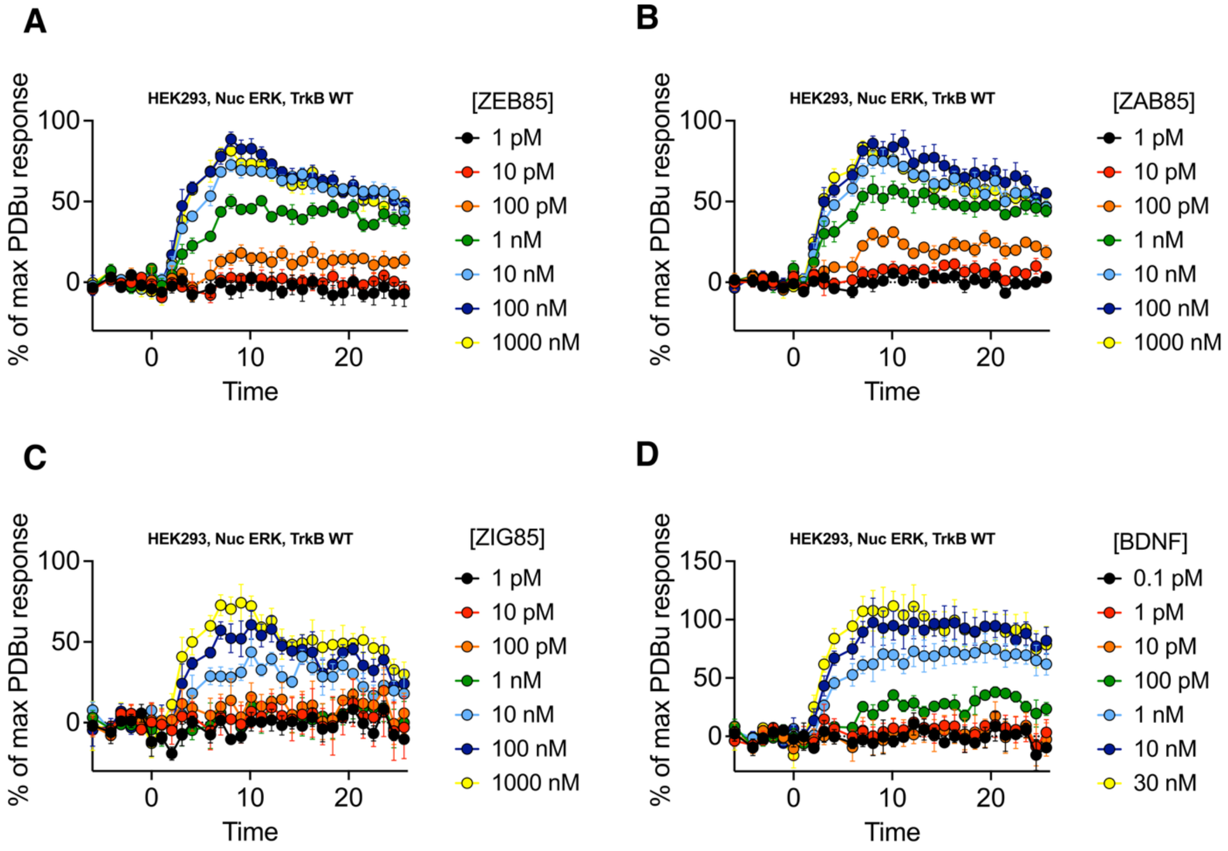

Fig. S3:

Real-time ERK signalling in response to ZEB85, ZAB85, ZIG85 or BDNF' and Human TrkB expressed in HEK293 cells alongside a nuclear ERK FRET sensor were stimulated with ZEB85 (A), ZAB85 (B), ZIG85 (C) or BDNF (D). Data from 4-5 independent replicates with triplicate wells. Mean ± SEM.

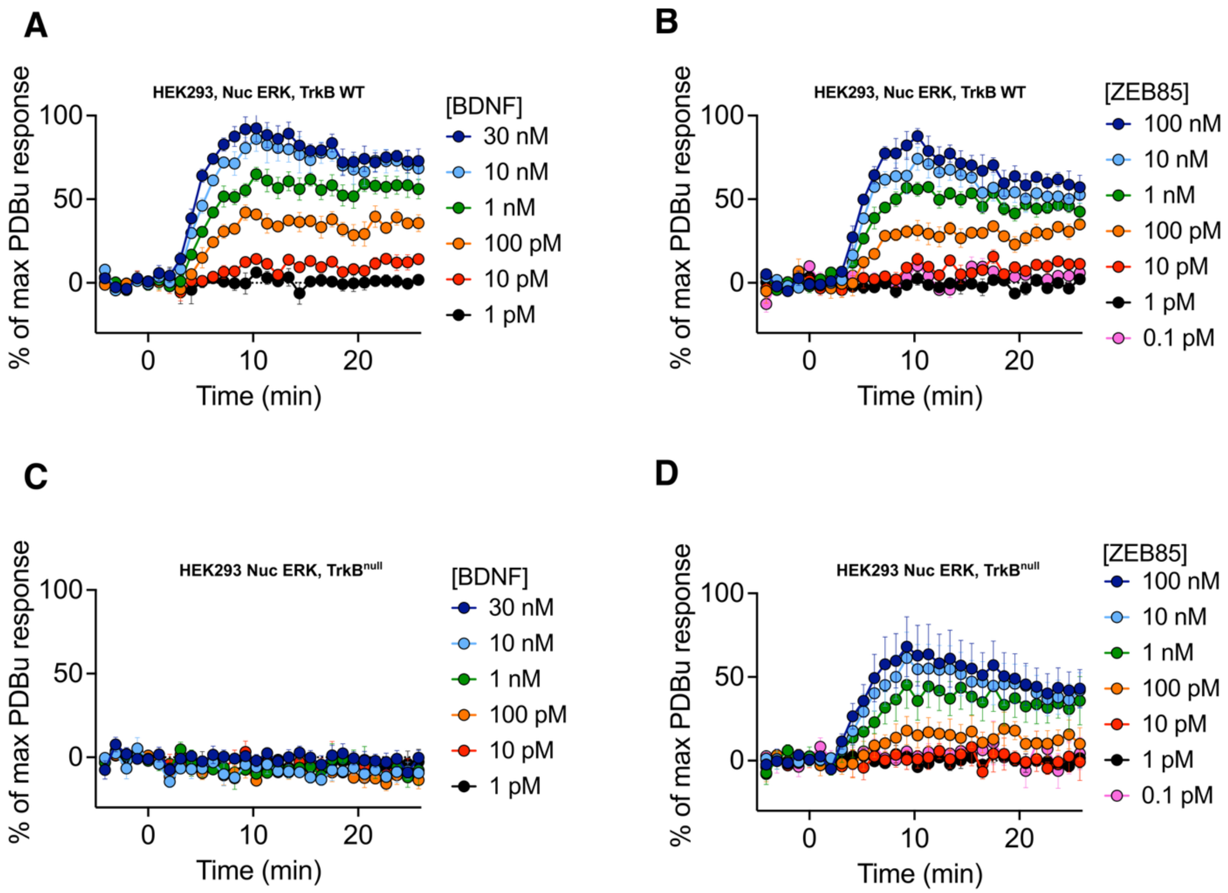

Fig. S4

TrkB[null] disrupted BDNF-induced ERK signalling, but not ZEB85-induced responses. Comparison of ERK responses to TrkB WT (A,B) or TrkB[null] (D298R/H299E/M379R) (C,D) in response to increasing concentrations of BDNF or ZEB85. Data from 4-5 independent replicates with triplicate wells. Mean ± SEM.

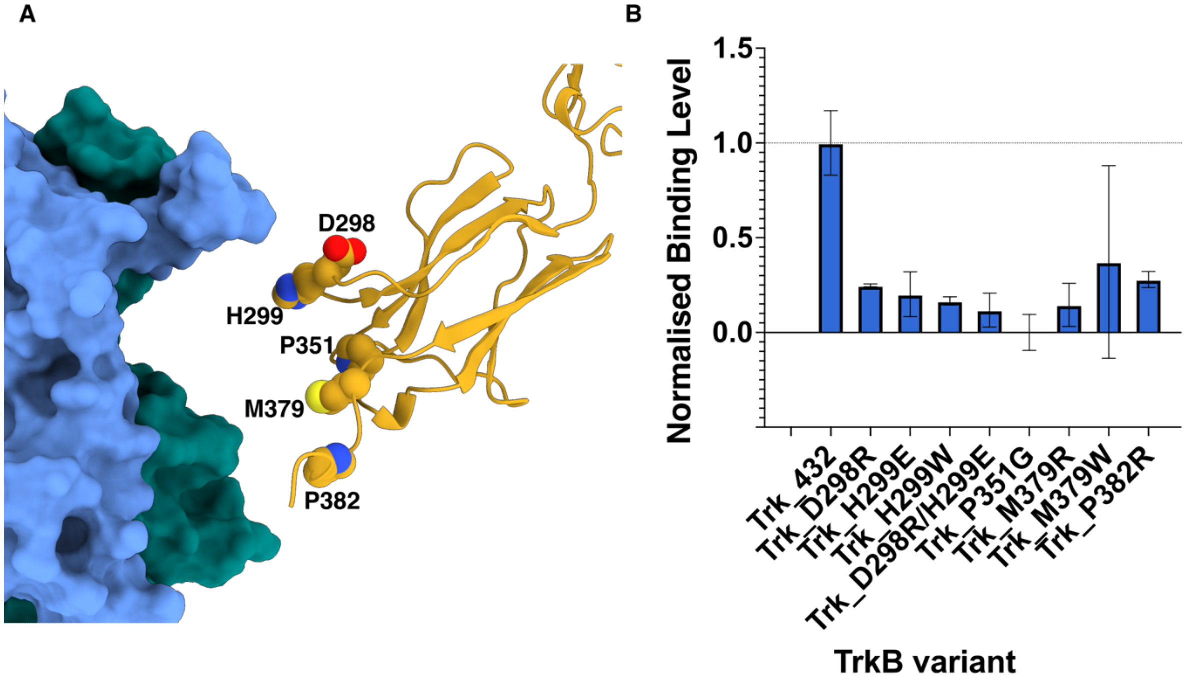

Fig. S5.

Design of a BDNF-binding deficient TrkB mutant, TrkB^{null}. (**A**) The sites of chosen mutations mapped onto our TrkB^{383}:BDNF^{mat} structure, (interface opened for clarity) (**B**) SPR binding analysis of TrkB variants. Mean of 3 experiments ± S.D. Values are normalized to wild type TrkB^{432}.

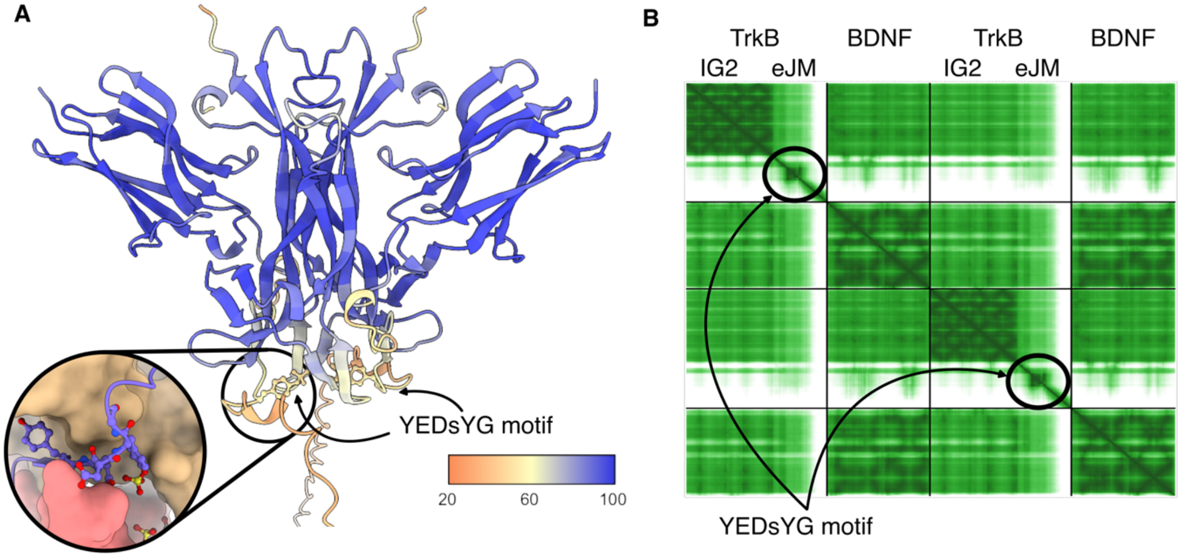

Fig. S6.

Alphafold 3 models of the TrkB/BDNF interface suggest a role for the residues 383 to 405 of TrkB eJM in binding BDNF. (**A**) The AlphaFold 3 model presented in Fig 3L and 3M coloured by pLDDT. Blue corresponds to high confidence, red corresponds to low confidence. Inset, shows a close-up of the predicted position of YEDsYG, occluded by BDNF. (**B**) Predicted aligned error plot for the same model. As before, the YEDsYG motif is highlighted.

Table S1.

	ZEB85 VHL Apo<br>PDB: 3OPO	ZEB85 VHL Bound<br>PDB: 3OPP	TrkB:BDNF complex<br>PDB: 3OPU
Wavelength	0.9795 Å	0.6199 Å	0.9796 Å
Resolution range	55.28 - 1.88<br>(1.94 - 1.88)	42.59 - 2.06<br>(2.13 - 2.056)	64.19 - 3.0<br>(3.11 - 3.0)
Space group	P 64 2 2	P 64 2 2	C 1 2 1
Unit cell	a=b=110.56<br>c= 184.30<br>90 90 120	a=b= 110.96<br>c= 184.01<br>90 90 120	a= 56.878<br>b=179.73 c=183.45<br>90 90.04 90
Total reflections	6541514 (609132)	3194219 (323105)	506973 (45341)
Unique reflections	54723 (5352)	42061 (4109)	36805 (3618)
Multiplicity	119.5 (113.8)	75.9 (78.6)	13.8 (12.5)
Completeness (%)	99.93 (100.00)	99.72 (99.47)	69.96 (25.06)<br>(*STARANISO processed*)
Mean I/sigma(I)	21.55 (0.31)	11.80 (0.59)	6.35 (0.09)
Wilson B-factor	48.46	47.87	107.63
R-pim	0.01209 (0.6188)	0.02689 (0.3572)	0.05366 (4.401)
CC1/2	1 (0.425)	1 (0.316)	0.982 (0.179)
CC*	1 (0.772)	1 (0.693)	0.995 (0.552)
Reflections used in refinement	54687 (5352)	42039 (4109)	25738 (912)
Reflections used for R-free	2669 (270)	2102 (201)	1349 (42)
R-work	0.1934 (0.3914)	0.2136 (0.3887)	0.2531 (0.4131)
R-free	0.2317 (0.4213)	0.2494 (0.3766)	0.2835 (0.4426)
Number of non-hydrogen atoms	3803	3844	7409
macromolecules	3550	3714	7311
ligands	94	71	98
solvent	207	101	0
Protein residues	459	478	936
RMS(bonds)	0.012	0.003	0.003
RMS(angles)	1.09	0.61	0.61
Ramachandran favored (%)	96.45	96.09	92.53
Ramachandran allowed (%)	3.55	3.91	7.14
Ramachandran outliers (%)	0.00	0.00	0.32
Rotamer outliers (%)	0.26	0.25	0.84
Clashscore	4.11	2.60	3.83
Average B-factor	61.09	63.62	106.01
macromolecules	61.02	63.82	105.38
ligands	69.46	68.22	152.85
solvent	60.38	55.20	N/A

X-ray Crystallographic data and refinement statistics
Statistics for the highest-resolution shell are shown in parentheses.

Table S2

Immobilised Partner	Immobilisation method	Analyte	K_D*
**TrkB_383**	Amine Coupling	ZEB85	N.B
**TrkB_432**	"	ZEB85	0.24 nM ± 0.08 nM
**TrkB_383-432 (Y400)**	Biotin/Streptavidin	ZEB85	2.34 µM ± 0.91 µM
**TrkB_393-405 (Y400)**	"	ZEB85	N.B
**TrkB_393-405 (sY400)**	"	ZEB85	0.52 nM ± 0.71 nM
**BDNF**	Amine Coupling	TrkB_432	9.8 nM ± 6.24 nM
**"**	"	TrkB_421	12.1 nM ± 1.15 nM
**"**	"	TrkB_412	3.8 nM ± 0.49 nM
**"**	"	TrkB_402	9.1 nM ± 0.19 nM
**"**	"	TrkB_393	37.7 nM ± 32.5 nM
**"**	"	TrkB_383	35.7 nM ± 18.4 nM
**"**	"	TrkB_432_D298R_H299E	N.B
**"**	"	TrkB_432_Y400F	15.3 nM ± 6.6 nM

Summary of Surface Plasmon resonance data

*Geometric mean ± 0.5x range, all SPR experiment n=3 or greater.

N.B = no binding detected.
